# Process evaluations of early childhood obesity prevention interventions delivered via telephone or text messages: a systematic review

**DOI:** 10.1186/s12966-020-01074-8

**Published:** 2021-01-09

**Authors:** Mahalakshmi Ekambareshwar, Swathi Ekambareshwar, Seema Mihrshahi, Li Ming Wen, Louise A. Baur, Rachel Laws, Sarah Taki, Chris Rissel

**Affiliations:** 1 Sydney School of Public Health, Faculty of Medicine and Health, The University of Sydney, Sydney, Australia; 2 NHMRC Centre of Research Excellence in the Early Prevention of Obesity in Childhood, Sydney, Australia; 3 School of Medicine and Public Health, University of Newcastle, Newcastle, Australia; 4 Department of Health Systems and Populations, Faculty of Medicine, Health and Human Sciences, Macquarie University, Sydney, Australia; 5 Health Promotion Unit, Population Health Research and Evaluation Hub, Sydney Local Health District, Sydney, Australia; 6 Faculty of Medicine and Health, The University of Sydney, Sydney, Australia; 7 School of Exercise and Nutrition Sciences, Institute for Physical Activity and Nutrition, Deakin University, Geelong, Australia

**Keywords:** Childhood obesity prevention, Telephone, Text messages, SMS, Apps, Antenatal/postnatal women, Caregivers, Systematic review, Early childhood, Process evaluation

## Abstract

**Background:**

Increasingly, public health interventions are delivered via telephone and/or text messages. Recent systematic reviews of early childhood obesity prevention interventions have not adequately reported on the way interventions are delivered and the experiences/perceptions of stakeholders. We aimed to summarise the literature in early childhood obesity prevention interventions delivered via telephone or text messages for evidence of application of process evaluation primarily to evaluate stakeholders’ acceptability of interventions.

**Methods:**

A systematic search of major electronic databases was carried out using the Population, Intervention, Comparison, Outcomes framework. Studies were included if interventions were delivered via telephone/text messages; aimed at changing caregivers’ behaviours to prevent early childhood obesity; with one or more outcomes related to early obesity risk factors such as breastfeeding, solid feeding, tummy time, sleep and settling, physical activity and screen time; published from inception to May 2020. All eligible studies were independently assessed by two reviewers using the Cochrane Collaboration tool for assessing risk of bias. Qualitative studies were assessed using the Consolidated Criteria for Reporting Qualitative Research and Standards for Reporting Qualitative Research tools.

**Results:**

Twenty-four studies were eligible, and the overall risk of bias was low. Eight studies (33%) had evidence of process evaluation that examined participants’ perceptions of interventions. Participants appreciated the convenience of receiving interventions via telephone or text messages. 63% of all studies in this review showed improvement in one or more behaviours related to childhood obesity prevention. Participants were likely to modify behaviours if they received information from a credible source such as from health professionals.

**Conclusion:**

There is limited reporting of stakeholders’ experiences in early obesity prevention studies delivered by telephone or text messages. Only one-third of studies examined participants’ acceptability and the potential for delivery of childhood obesity prevention interventions conveniently using this mode of delivery. Interventions delivered remotely via telephone or text messages have the potential to reach equal or a greater number of participants than those delivered via face-to-face methods. Future research should build in process evaluation alongside effectiveness measurements to provide important insight into intervention reach, acceptability and to inform scale up.

**Trial registration:**

PROSPERO registration: CRD42019108658

## Background

The high prevalence of obesity is recognised world-wide, with an increasing interest in the prevention of obesity in the early years, from pre-birth up to and including 5 years of children’s age. Early childhood obesity prevention has gained momentum during the last decade, with a focus on children’s primary carergivers, mothers in most instances, as key agents to whom interventions are delivered [1–4]. Early prevention studies have utilised existing platforms such as mothers’/parents’ groups [3], child health clinics [4] and post-birth follow-up home visits by nurses [2] to deliver key messages to caregivers.

There has been an exponential growth of mobile phone ownership and its use globally, both in developed and developing countries alike [5, 6]. In Australia alone, an estimated 92% over the age of 18 used a mobile phone in 2012, additionally over half of those aged 25–34 were mobile-only phone users [7]. Public health and health promotion researchers have harnessed the increased dependability on mobile phones to deliver interventions via telephone and/or text messages [8, 9]. Crucially, this mode of delivery was welcomed for its cost-effectiveness [10], ability to reach wider population [11] and its acceptability to those receiving the interventions [12].

Population-wide increases in communication via telephone and/or text messages has led to growth in the number of interventions delivered using these modes in clinical care, public health and health promotion. Earlier examples have included text messages to patients to send medical appointment reminders [13], text messages for routine chronic disease management [14, 15], and telephone calls for mental health management [16]. There has also been extensive use of telephone calls and/or text messages by public health and health promotion researchers to communicate health promotion messages and public health interventions [17, 18]. Similarly, there has been a growth in the number of studies using mobile phones to communicate key messages to new caregivers and women with young children [1, 19, 20].

To date, findings of systematic reviews of telephone and text message support have suggested improved outcomes among several groups: in pregnant women and new mothers who received telephone support for smoking, breastfeeding, birthweight and postpartum depression [21]; in adults who received telephone-delivered interventions for physical activity and dietary outcomes [22]; in pregnant women who received telephone support for depression and breastfeeding during pregnancy and post-birth [23]; and in adolescents who received text message interventions for physical activity and sedentary behaviours [24]. Interventions for childhood obesity prevention or behaviour change delivered via telephone or text messages and their effectiveness have been established and reported, however process evaluation among study participants as well as stakeholders is often less well reported [25, 26].

In this systematic review, we aimed to examine early childhood obesity prevention interventions delivered via telephone or text messages (solely or supplementary to traditional modes), for evidence of process evaluation. Our objective was to explore the acceptability of the interventions to stakeholders, primarily to participants, intervention deliverers, health managers and policymakers.

## Methods

This systematic review adhered to the Preferred Reporting Items for Systematic Reviews and Meta-Analysis (PRISMA) standardised reporting guidelines and checklist [27].

### Protocol and registration

A protocol was developed prior to the review process and was registered with the International Prospective Register for Systematic Reviews (PROSPERO). It can be accessed via (https://www.crd.york.ac.uk/prospero/ registration number: CRD42019108658).

### Eligibility, study inclusion and exclusion criteria

Eligible studies were identified using the Population, Intervention, Comparison, Outcomes (PICO) framework [28]. Patient Problem (or Population) – pregnant women or caregivers who received childhood obesity prevention interventions for children from birth up to and including 5 years of age. Intervention – interventions aimed at changing caregivers’ behaviours to prevent early childhood obesity; delivered via telephone (including via telephone applications (apps)) or text messages primarily or supplementary to face-to-face or online methods. Comparison or control – caregivers who received usual care or maintenance care (for example, control group in randomised controlled trials (RCT), non-equivalent control group in quasi-experimental design). Outcome – one or more early obesity prevention or behaviour change outcomes such as body mass index (BMI), breastfeeding, solid feeding, “tummy time” (allowing babies time lying prone on their abdomen while they are awake), sleep and settling, physical activity, screen time and participant well-being.

The review encompassed intervention studies including randomised and cluster-randomised controlled trials, case control studies, quasi-experimental studies without comparators and descriptive studies with evidence of program outcome(s). The review included studies that delivered interventions via telephone (including apps) or text messages (solely or supplementary to traditional modes). We focussed specifically on those studies undertaking process evaluation to explore participant and health professional experiences. Studies were excluded if they did not have at least one childhood obesity related or behaviour change outcome, and if studies only reported outcomes of children older than 5 years of age.

### Information sources

The following databases were searched from their inception to 15 May 2020, to identify eligible trials: MEDLINE (OVID; 1966), Scopus (Elsevier 1980), Web of Science (Clarivate Analytics post-2016, Thomson Reuters pre-2016); CINAHL Complete (EBSCOhost; 1994), the Cochrane Library databases, Database of Systematic Reviews, and the US National Library of Medicine’s ClinicalTrials.gov. We also searched the reference lists of several relevant systematic and narrative reviews, grey literature including doctoral theses and conference proceedings, relevant government websites, Google Scholar and Google Search.

### Search

Preliminary literature searches were carried out in 2018 to assess the feasibility of the review. The full electronic search strategy is provided in Table 1. A comprehensive literature search was conducted by one author (ME) in May 2019 and repeated in May 2020.

**Table 1:** Searches on MEDLINE, SCOPUS, WEB OF SCIENCE and CINAHL from inception to May 2020

		Number of records Search updated 15/5/2020	Number of records Search conducted 23/5/2019
**For MEDLINE (OVID; 1966)**			
1	exp infant/	1,128,327	1,096,984
2	child, preschool/ or exp. infant, newborn/	1,346,166	1,306,248
3	child*.tw.	1,362,646	1,297,657
4	p?ediatr*.tw.	345,746	321,730
5	newborn*.tw.	159,763	154,057
6	toddler*.tw.	10,743	9884
7	pre?school*.tw.	28,089	26,452
8	babies.tw.	36,506	35,004
9	baby.tw.	37,467	35,675
10	neonat*.tw.	260,610	248,563
11	infan*.tw.	432,073	414,324
**12**	**or/1–11**	**2,646,778**	**2,540,388**
13	pediatric obesity/	8130	6686
14	p?ediatric obesity.tw.	1327	1226
15	obesity/	177,751	169,076
16	obes*.tw.	292,628	272,156
17	overweight/	24,244	22,316
18	over?weight.tw.	67,592	62,394
19	over?fe*.tw.	2025	1912
20	weight gain/ or weight loss/	64,821	61,683
21	(weight adj4 (loss or gain or excess or increase or decrease)).tw.	171,307	161,199
22	BMI.tw.	139,870	127,847
23	Body mass index.tw.	178,809	164,850
24	body mass index/	124,645	117,367
**25**	**or/13–24**	**642,516**	**600,221**
26	exp health promotion/	75,732	72,135
27	exp health education/	241,031	232,601
28	(health* adj4 (behavio?r or promot* or educat* or eat* or food*)).tw.	171,123	157,573
29	behavio?r therapy.tw.	6652	6336
30	early intervent*.tw.	18,692	17,152
31	early child*.tw.	26,789	24,884
32	motivat*.tw.	129,595	119,624
33	exp child health services/	24,033	23,215
34	social support*.tw.	38,032	35,119
35	counsel*.tw.	106,490	99,853
36	(parent* adj3 group*).tw.	7327	6943
37	mother* group*.tw.	340	325
38	breastfeeding/	37,064	35,511
39	breast?feed*.tw.	25,812	23,688
40	breast?fed*.tw.	6694	6203
41	((infant* or child*) adj4 (feed* or food* or meal* or diet*)).tw.	35,522	33,342
42	(feeding adj4 (practice* or behavio?r* or style*)).tw.	18,344	17,186
43	solid*.tw.	352,781	328,752
44	(introduc* adj3 solid*).tw.	1389	1292
45	diet*.tw.	552,787	522,485
46	nutr*.tw.	397,898	370,735
47	(diet* adj4 (intake or modification* or habit*)).tw.	66,999	62,921
48	vegetable*.tw.	54,058	50,178
49	fruit*.tw.	104,890	96,805
50	eating habit*.tw.	5276	4862
51	play*.tw.	1,176,268	1,102,335
52	exp exercise/	191,577	178,510
53	physical activit*.tw.	108,536	99,211
54	((screen* or device* or computer* or television* or TV) adj4 time).tw.	18,403	16,937
55	sedentar*.tw.	31,004	28,619
56	supine* position.tw.	10,881	10,400
**57**	**or/26–56**	**3,247,052**	**3,048,762**
58	pregnant women/	8099	7443
59	(pregnan* adj4 (wom?n or mother*)).tw.	140,528	131,818
60	((ante?natal or pre?natal or post?natal or post?partum or post?birth) adj4 wom?n).tw.	17,970	16,561
61	(expec* adj3 mother*).tw.	1969	1833
62	mothers/	42,794	39,792
63	mother*.tw.	216,584	205,204
**64**	**or/58–63**	**347,003**	**327,952**
**65**	**12 and 25 and 57 and 64**	**8097**	**7401**
66	cell phone/ or text messaging/	10,432	9442
67	(mobile* or telephone* or phone* or smart?phone* or cell?phone* or hand?held).tw.	189,480	175,681
68	(text* adj2 messag*).tw.	4048	3474
69	SMS.tw.	5750	5204
70	(mobile* adj3 app*).tw.	7110	5779
**71**	**or/66–70**	**197,231**	**182,657**
**72**	**65 and 71**	**220**	**202**
**SCOPUS (Elsevier 1980)**			
search terms as in MEDLINE		**280**	**237**
**WEB OF SCIENCE (Clarivate Analytics post-2016, Thomson Reuters pre-2016)**			
search terms as in MEDLINE		**488**	**429**
**CINAHL Complete (EBSCOhost; 1994)**			
search terms as in MEDLINE		**4**	**4**

Free text terms searched

* truncation

# wildcard

? wildcard

adj adjacent

.tw textword field includes title and abstract

exp exploded term

### Study selection

Titles and abstracts of references were independently screened by two reviewers (ME and SE) in Covidence systematic review software (www.covidence.org). Disagreements were resolved by discussion with a third reviewer (SM), where necessary. Following the retrieval of full texts, the same two reviewers independently screened them against the specified inclusion/exclusion criteria defined above. Papers relating to the same trial were grouped into one study.

### Data collection process

Records from all databases and hand searches were imported or recorded into a reference management software package (Endnote version X9) and then exported from Endnote to Covidence. Duplicate records were removed. Any additional articles identified from reference lists of included trials were included to supplement the analysis.

### Data extraction

Data were extracted using a data extraction table that represented the categories of intended data items which were tested and piloted for feedback from all authors. After agreement was reached, ME extracted all data that were reviewed by at least one other author (Table 2). For those studies without reported outcomes, we contacted authors of the trials to obtain the required data.

**Table 2:** Studies included in this review

Trial name: First author publication year (year study commenced), Country, (Reference #)	Age of child at Intervention commencement (intervention duration) in months	Measured study outcomes	Study design	Main medium of intervention delivery	Supplementary medium of intervention delivery	Intervention delivery provider	Intervention setting	No. of sessions	Parity	Retention %	Measurement time points	Care giver age (years)	Qualifications of mother
INFANT: Campbell 2008 (2008), Australia [3]	3 (15)	BMI; TV viewing, PA and non-core drink & fruit, veg, dietary intake	Cluster-randomised controlled trial	Group sessions	Text messages + mail-outs	Dietitians	Parent groups	6 calls (number of text messages not specified)	Primiparous 100%	89	3 months, 9 months, 20 months	32.3	54.2% tertiary qualified
Carlsen 2013 (2010), Denmark [29]	At birth (6)	BMI; exclusive breastfeeding & any breastfeeding	Randomised trial	Telephone	None	Certified lactation consultant	Participant choice	9	Primiparous 67%	85	1 months, 3 months, 6 months	31.3	Not reported
PRIMROSE: Doring 2014 (2014), Sweden [30]	9 (48)	BMI and waist circumference; eating habits, physical activity	Cluster-randomised trial	Face-to-face	Group sessions + telephone	Child Health Centre nurses	Child Health Centres	9 total - 7 face-to-face; 2 telephone	First-time mothers, 100% primiparous	unclear	BMI at baseline (6–9 months); BMI at 4 years of child’s age;	30.3	66.8% tertiary qualified
Franco-Antonio 2018 (2018), Spain [31]	At birth (6)	Breastfeeding; breastfeeding self-efficacy	Multi-centre parallel group RCT	Combination: Face-to-face + telephone	None	Midwife or trained nurse	Community	4	Primiparous (34.1%)	92	0 mo (baseline), 1 month, 3 months, 6 months	32.8	26.1% tertiary qualified
MumBubConnect Gallegos 2014 (2010), Australia [32]	2.5 (2)	Breastfeeding rates and breastfeeding self-efficacy	Non-concurrent, prospective comparison trial	Text messages	Telephone + social media	Breastfeeding counsellor	Participant choice	8	Not reported	86	2–3 months, 4–5 months	31	61% tertiary qualified
Gibby 2019 (2019), Hawai’I & Puerto Rico [33]	Near birth (4)	Weight changes; feeding practices	Randomised controlled trial	Text messages	None	Third-party web-based text messaging platform	Participant choice	18	Primiparous (40.2%)	84	0-1mo and 4-6mo	27	53.5% tertiary qualified
Steps to Growing Up Healthy: Gorin 2014 (2014), USA [34]	35 (12)	BMI	Randomised trial	Combination: Face-to-face + telephone + home visits	None	Medical team (clinician, paediatric resident, nurse and medical assistant); phone calls and home visits by community health worker (CHW)	Paediatric primary care clinic	4	Not reported	90	35mo and 47mo	35.4	Not reported
Healthy Habits, Happy Homes: Haines 2013 (2011), USA [35]	48 (6)	Behavioural outcomes: eating meals together as family, sleep duration, TV viewing time, presence of TV in child’s sleeping room)	Randomised trial	Combination: Home visits + telephone + text messages + educational materials	None	Health educator	Mainly at home	48 total - 4 home visits; 4 telephone calls; 40 text messages	Not reported	92	48mo and 54mo	Not reported	49% tertiary qualified
Hannan 2012 (2012), USA [36]	At birth (2)	Weight gain	Randomised clinical trial	Telephone	None	Paediatric nurse practitioners	Participant choice	6	Primiparous 100%	unclear	0 and 2 months	24.1	69.1% high school qualified or more
Harris-Luna 2018 (2018), USA [37]	At birth (3)	Exclusive Breastfeeding and Breastfeeding duration	Pragmatic design	Telephone	None	Certified promotoras	Participant choice	8	Not reported	100	0 to 12 weeks	26.36	Not reported
M528: Hmone 2017 (2015), Myanmar [38]	During pregnancy 28 weeks’ gestation (9)	Exclusive breastfeeding rate	2-group parallel-arm randomised controlled trial	Text messages	None	Text messages were sent by CommConnect,Telerivet	Participant choice	117 text messages	Primiparous 57%	79	1,2,3,4,5 & 6 months	60% 25–34 age group	96% high school qualified
Healthy Babies: Horodynski 2011 (2011), USA [39]	1 (11)	Infant growth pattern; maternal knowledge and self-efficacy; feeding practices	Randomised experimental short-term longitudinal controlled trial	Home visits	Telephone	Paraprofessional instructor	Mainly at home	9	Not reported	unclear	1 month (baseline), 6 months and 12 months	Not reported	75% high school qualified
Jiang 2014 (2014), China [40]	3rd trimester of pregnancy (15)	Duration and rate of EBF; timing of intro of solid foods; other feeding practices (eg. cup feeding, bottle feeding, food reward)	Quasi-experimental design	Text messages	None	FrontlineSMS used for sending messages	Participant choice	66	Primiparous 100%	82	Breastfeeding at 4,6,12mo; Intro of solid foods at 4, 6mo; infant feeding behaviours at 12mo	25–29 age group (58.4%)	100% high school qualified
The Baby Milk Trial: Lakshman 2015 (2011), UK [41]	At birth (6)	Change in weight; change in BMI; skinfold thickness	Explanatory, parallel, individually randomised controlled trial	Combination: Face-to-face + telephone + leaflets + stickers	None	Research nurses	Health check clinics	5	Primiparous 51.9%	87	Baseline, 6 months and 12 months	31.9	59.3% tertiary qualified
Growing Healthy: Laws 2018 (2015), Australia [42]	At birth (9)	Breastfeeding duration; Intro to solids; formula preparation (where applicable)	Quasi-experimental design	Combination: App + website + push notifications + text messages	Emails + social media	App and website developed by research team	Participant choice	72 push notifications + text messages but App available 24 × 7	Primiparous 57.5%	80	Baseline (3mo), 6 months and 9 months	30.4	78.9% tertiary qualified
Smart Moms: Nezami 2018 (2014), USA [43]	55 (6)	Child SSB consumption and BMI	Two-group randomised controlled trial	Combination: Group session + Website + text messages	None	Principal investigator	Community group session	73 total - 1 group + 72 text messages	Not reported	82	Baseline (56.4 mo), 3 months and 6 months post-intervention	36.6	81.5% tertiary qualified
Patel 2018 (2010), India [44]	3rd trimester of pregnancy (9)	Exclusive Breastfeeding; bottle feeding	Two-arm, hospital-based pilot study	Combination: Telephone + text messages	None	Auxiliary nurse midwives	Participant choice	288 total - 36 calls + 252 text messages	Not reported	92	24 h after birth, weeks 6, 10 14 and at 6 months	<=24 age group 50.9%	29.5% high school qualified
WIC: Pugh 2010 (2003), USA [45]	At birth (6)	Breastfeeding	Two-group randomised controlled trial	Hospital and home visits	Telephone	Nurse + Peer counsellor	Mainly at home	17	Primiparous 50.6%	71	6 weeks, 12 weeks and 24 weeks postpartum	23.1	11.3% tertiary qualified
Tahir 2013 (2010), Malaysia [46]	At birth (6)	Breastfeeding practice	Single blinded randomised controlled trial	Telephone	None	Certified lactation counsellors (Nurses trained in lactation counselling)	Participant choice	12	Primiparous 38.1%	89	1 month, 4 months, 6 months	28.6	27.7% tertiary qualified
Baby’s first bites: van der Veek 2019 (2019), Netherlands [47]	4 (12)	Vegetable consumption, vegetable liking, child eating behaviours, child anthropometrics	Four-arm randomised controlled trial	Combination: Print materials + telephone	None	Trained researchers or Master’s students	Participant choice	5	100%	95	Baseline, 18, 24 and 36 months	30.4	41.6% high school qualified
Mothers & Others: Wasser 2017 (2013), USA [48]	At 30 weeks’ gestation during pregnancy (14)	Mean weight-for-length z-score; exclusive breastfeeding; intro to complementary foods; fruit and vegetable intake; infant sleep; and expsoure to television	2-group randomised controlled trial	Combination: Home visits+ Newsletters+ text messages	None	Peer Educator plus Certified Lactation Consultant	Mainly at home	Total 130–8 home visits + 122 text messges	Not reported	unclear	Not reported	Not reported	Not reported
Healthy Beginnings: Wen 2007 (2007), Australia [2]	At 30 weeks’ gestation during pregnancy (26)	Duration of breastfeeding; delayed introduction of solids; feeding habits; tv viewing; active play; overweight and obesity	Randomised controlled trial	Home visits	Telephone	Specially trained community nurse	Mainly at home	8 plus pro-active telephone	Primiparous 100%	75	Baseline, 6 months; 12 months; 24 months	26	80.4% high school qualified
CHAT: Wen 2017 (2017), Australia [1]	At 28 weeks’ gestation during pregnancy (15)	Tummy time, drinking water, cup feeding, TV viewing, BMIz, TV viewing, fruit / vegetable intake	3-arm parallel randomised controlled trial	Combination: Telephone + intervention booklets (or) text messages + intervention booklets	Email	Child and family health nurse (telephone); automated SMS	Participant choice	6 calls or 48 text messages	Primiparous 54%	80	Baseline (on recruitment); 6 months; 12 months	< 30 age group 70%	68% Tertiary qualified
Linked trial for HB: Wen 2019 (2019), Australia [19]	24 (12)	BMI and BMIz; diet, PA and screen time; cost-effectiveness	Parallel randomised controlled trial	Combination: Telephone + text messages + intervention booklets	Email	Child and Family Health Nurse/midwife	Participant choice	3 calls plus 48 text messages	Ongoing	ongoing	Baseline at 24 months and 36 months of child’s age	Ongoing	Ongoing

### Process evaluation

We analysed all eligible studies (and associated published literature) that described process evaluation or assessed program satisfaction through quantitative and/or qualitative surveys. Although process evaluation includes several components, we focussed on stakeholders’ perceptions of interventions that are fundamental to their subsequent implementation and effectiveness. Some process evaluation measures that we explored included continued participation (retention), ease and convenience of delivering interventions (feasibility), acceptability of interventions by participants, adherence to advice provided, and experiences of participants, intervention deliverers and researchers.

### Planned methods of analysis

Comprehensive analysis of all eligible studies (and related published literature) was undertaken to identify studies that conducted process evaluation. We gathered and analysed data informed by the Template for Intervention Description and Replication (TIDieR) [49]. The data included name, theoretical framework, what interventions were delivered, who delivered the interventions, how (mode of delivery), where the interventions were delivered (intervention setting), number of times and over what period the interventions were delivered or dose (number of sessions and frequency of intervention delivery), and intervention adherence or fidelity (retention). Additionally, we gathered data relevant to this review such as design, objectives, outcomes, parity or birth order. For synthesis of process evaluation data, a convergent segregated approach [50, 51] was used to firstly enable synthesis of quantitative and qualitative evidence within studies, followed by narrative synthesis to determine the experiences / perceptions of participants and health professionals (where available) who received or delivered the interventions [50, 52]. For ongoing studies, we tried to contact the study investigator where possible to obtain further information.

### Risk of bias in individual studies

All eligible studies were independently assessed by two reviewers (ME and SE) using the Cochrane Collaboration tool for assessing risk of bias [53]. Disagreements were resolved through discussion with a third reviewer, when necessary. Studies that met the eligibility criteria were assessed for all five domains, namely, randomization process, deviations from intended interventions, missing outcome data, measurement of outcome, and selection of the reported result [53]. Risk was reported as ‘high’ or ‘low’ or ‘some concerns’, as recommended in the Cochrane Risk of Bias (RoB 2) revised tool [54].

### Assessment of qualitative studies

While risk of bias assessment enables confidence that estimates of effect are near true values for outcomes, it does not assess the qualitative inquiry [53]. Therefore, eligible qualitative studies that demonstrated evidence of process evaluation, satisfaction or feasibility measures were assessed for rigour to investigate the extent to which study authors conduced their research to the highest possible standards. Studies were assessed against the Consolidated Criteria for Reporting Qualitative Research (COREQ) [55] and the Standards for Reporting Qualitative Research (SRQR) checklists [56]. COREQ and SRQR include 32-item and 21-item checklists, respectively, that draw together important aspects of qualitative research to assess the reporting of relevant information. There are three broad domains in COREQ: research team and reflexivity (personal characteristics, relationship with participants); study design (theoretical framework, participant selection, setting, data collection); and analysis and findings (data analysis, reporting). In SRQR, the first two items are the article’s title and abstract; the remaining 19 items relate to congruity between authors’: problem formulation and research question; research design and methods of data collection and analysis; results, interpretation, discussion, and integration; and other information.

## Results

### Study selection

**Fig. 1:**
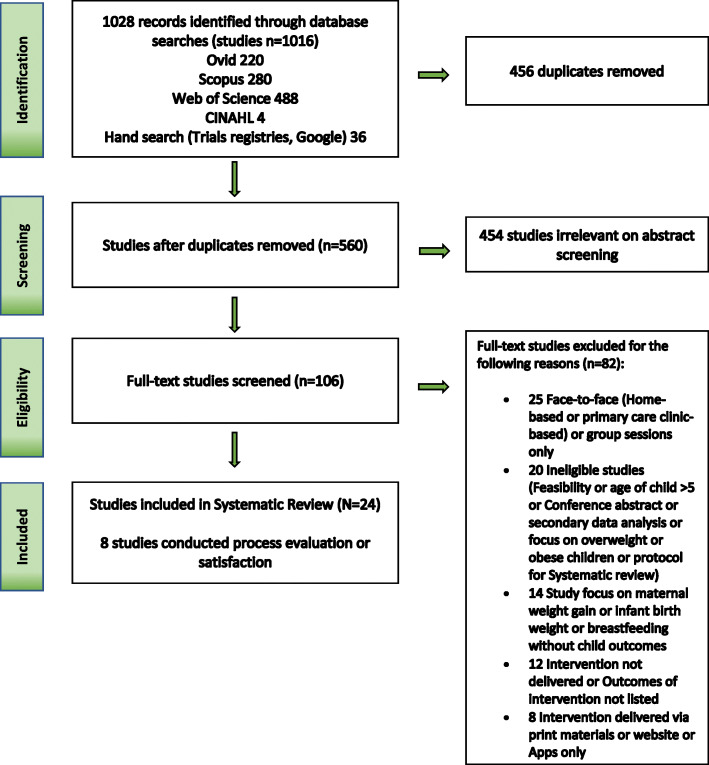
Preferred Reporting Items for Systematic Reviews and Meta-Analyses (PRISMA) flowchart screening of literature search and eligibility

We identified 1028 records after the systematic conduct of electronic and hand searches. After duplicate removal, title and abstract screening, 106 references were selected for full-text review. Twenty-four studies were finally included in this review (Fig. 1). A list of included studies is provided in Table 2.

### Characteristics of studies

Key study characteristics are presented below and described in detail in Table 3.

**Table 3:** Study characteristics

Characteristic	Category	Studies	
		n	%^a^
**STUDY**			
Year study commenced (*N* = 24)	2003 to 2011	5	21
	2012 to 2016	7	29
	2017 to 2019	12	50
Country of studies (*N* = 24)	Australia	6	25
	China	1	4
	Denmark	1	4
	India	1	4
	Malaysia	1	4
	Myanmar	1	4
	Netherlands	1	4
	Spain	1	4
	Sweden	1	4
	UK	1	4
	USA	9	38
Study design (*N* = 24)	RCT	18	75
	Cluster RCT	2	8
	Non-concurrent, prospective comparison trial	1	4
	Pragmatic design	1	4
	Quasi-experimental design	2	8
Participant retention rate (*N* = 24)	> 90%	6	25
	80–90%	9	38
	< 80%	4	17
	Not specified/unclear/ongoing	5	21
**MEDIUM**			
Intervention setting (*N* = 24)	Community settings (Child health centres, parent groups, health check clinics, primary care)	6	25
	Home	5	21
	Participant choice	13	54
Mode of intervention delivery (*N* = 24)	Face-to-face (group or home visits or community settings) + Supplementary telephone	7	29
	Face-to-face (group or home visits or community settings) + Supplementary text messages	3	12
	Face-to-face (group or home visits or community settings) + Supplementary telephone and text messages	1	4
	Telephone	5	21
	Telephone (incl. apps) + Text messages	5	21
	Text messages	3	12
**POPULATION**			
Age of child at intervention commencement (*N* = 24)	Prior to child’s birth (during pregnancy)	6	25
	0–3 months	10	42
	4–6 months	3	12
	7–9 months	1	4
	> 12 months	4	17
Mean age of mother (*N* = 20)	21–30 years	12	60
	31–40 years	8	40
Parity of mothers (*N* = 14)	Primiparous 100%	5	36
	Primiparous 50–99%	6	43
	Primiparous < 50%	3	21
Qualifications of mother (*N* = 18)	Tertiary (> 50%)	9	50
	Tertiary (< 50%)	2	11
	High school (> 50%)	5	28
	High school (< 50%)	2	11
**INTERVENTION**			
Intervention duration (*N* = 24)	≤ 6 months	11	46
	7–12 months	7	29
	13–24 months	4	17
	> 24 months	2	8
Intervention deliverers (*N* = 24)	Automated	5	21
	Counsellor	1	4
	Dietitians	2	8
	Health Educator/Instructor	4	17
	Lactation consultant	1	4
	Medical Team	1	4
	Nurse + Peer Counsellor	1	4
	Nurses / Midwives	9	38
Number of intervention sessions			
Face-to-face ± telephone	1 to 9	16	
	10 to 19	2	
	> 20	1	
Text messages	1 to 9	1	
	10 to 19	1	
	20 to 49	3	
	> 50	6	
**OUTCOMES**			
Number of outcomes measured (*N* = 24)	One	6	
	Two	4	
	Three	9	
	Four or more	5	
Child outcomes measured	BMIz	13	
	Weight gain	3	
	Breastfeeding	16	
	Solid feeding / food habits	15	
	Tummy time	3	
	Play time / Physical activity	5	
	Sleep duration / sleep quality	4	
	TV viewing / Screen time	7	
	Goal setting for mothers	4	
	Mother’s well-being	2	
Measurement time points (child’s age)	0–3 months	24	
	4–6 months	16	
	7–9 months	3	
	10–12 months	5	
	12–24 months	4	
	> 24 months	9	
**PROCESS EVALUATION / SATISFACTION**			
Process evaluation / satisfaction (*N* = 24)	Mention of process evaluation	8	33
	Mention of Satisfaction measure	3	12
Evaluation post-intervention	Quantitative survey	1	
	Qualitative interviews	5	
Evaluation during intervention	Quantitative survey	8	
	Qualitative interviews	4	

Abbreviations: RCT randomised controlled trial; UK United Kingdom; USA United States of America

^a^Numbers rounded so total may not add up to 100

### Study design and participation rates

The majority of identified studies (19 out of 24) were published in the last decade, of which one-half were published within the last 4 years. Sixty-three percent of studies were conducted in the USA or Australia. The majority (80%) were RCTs, of which 18 were individual RCTs and two were cluster RCTs; two had a quasi-experimental design and the remaining two studies were pragmatic. Key study characteristics are represented in Table 3. Retention rates ranged from 71 to 100%, and 16 studies (67%) indicated participant retention rates of greater than 80%.

### Setting and medium of intervention delivery

More than half (54%) of the studies (13 of 24) delivered interventions exclusively and flexibly via telephone and/or text messages where participants or deliverers did not need to go to a predetermined location to receive or deliver interventions. The remainder were face-to-face sessions, group sessions or home visits supplemented by telephone or text messages.

### Target population

Interventions were delivered to caregivers who were predominantly women. Intervention delivery commenced as early as when women were pregnant (25%), as well as when the children were < 3 months of age (42%), 4–12 months of age (16%) and > 12 months of age (17%). In studies where the mean age of participants was reported (*n* = 20), the majority (60%) were aged 30 years or under. Parity was reported by 14 studies (58%); five of these studies delivered interventions to first-time mothers only.

### Intervention characteristics

Almost one-half of the studies (46%) delivered interventions for a period of 6 months or less, 29% delivered interventions for a period of 7–12 months, 17% delivered interventions for a period of 13–24 months, while 8% delivered interventions for longer than 24 months. Interventions were delivered via nurses or midwives (38%), health educators (17%), dietitians (8%), or automated text messages, apps or online (21%).

### Intervention components

Interventions were delivered for breastfeeding, food and drink intake, “tummy time” (allowing babies time lying prone on their abdomen while they are awake), play time / physical activity, sleep, screen time, goal-setting and maternal well-being. The number of outcomes measured typically varied between one and four, with most studies reporting fewer than four outcomes.

### Risk of bias within studies

**Fig. 2:**
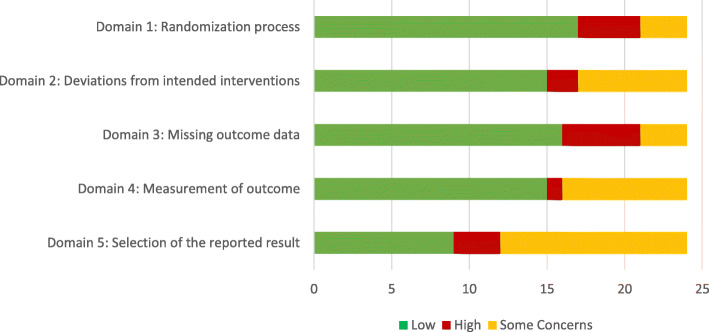
Risk of bias assessment of eligible studies (*N* = 24)

We included all types of studies in this review, hence in the domain ‘randomisation process’ four studies were judged as having ‘some concerns’ as they did not randomise participants or lacked adequate information on the randomisation process. For the domain ‘deviation from intended outcomes’, seven studies were judged as having ‘some concerns’ as they did not provide adequate information on the blinding of participants and intervention deliverers. Nineteen studies had high participant retention rates (> 70%) and were judged as low risk; five had low participant retention which were assessed as high risk in the ‘missing outcomes data’ domain. Information on ‘measurement of outcome’ was provided clearly by 16 studies, the remaining 8 studies that lacked adequate information or were ongoing were judged as having ‘some concerns’. Eleven studies in this review stated that more than one outcome and/or outcomes were measured at various time points; therefore, in the domain ‘reporting of the selected results’, studies without published evidence of outcomes at the various time points were judged as having ‘some concerns’. Risk of bias is represented in Fig. 2. Full details of our assessment of bias are in Table 4. Five studies had low risk of bias in all five domains.

**Table 4:**
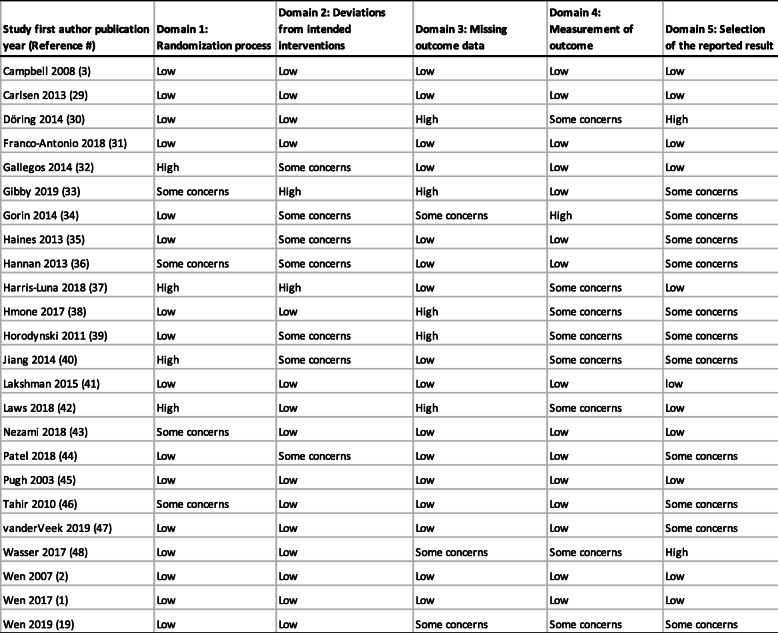
Detailed risk of bias assessment of eligible studies (*N* = 24)

### Outcomes / effectiveness of studies

There were 24 eligible studies in this review, with details of outcomes of studies provided in Table 5. Sixteen studies measured anthropometric outcomes of which less than a quarter reported statistically significant age appropriate lower BMI z-score (BMIz) in the intervention group in comparison to the control group. Thirteen studies measured age appropriate BMIz [1–3, 19, 29, 30, 33–35, 40, 42, 43, 47] and three measured age appropriate weight gain in children as an outcome [36, 41, 48] . Sixteen studies measured duration of breastfeeding [1, 2, 19, 29, 31–33, 37–40, 42, 44–46, 48]; 15 studies reported on solids feeding or food habits of the children [1–3, 19, 30, 33, 34, 38–43, 47, 48]; 3 studies reported on the practice of tummy time [1, 2, 19]; 5 studies reported on play time / physical activity in children [1, 2, 19, 34, 58]; 4 studies reported on sleep duration / sleep quality [1, 2, 19, 35]; and 7 studies reported on children’s screen time/ television (TV) viewing time [1–3, 19, 34, 35, 48] (Table 5).

**Table 5:** Outcomes/effectiveness of interventions

Trial name: First author publication year (year study commenced), Country (Reference #)	Outcomes measured (in comparison to control)								
	BMIz	Breastfeeding changes	Solid feeding / feeding habit changes	Tummy time	Play time and/or physical activity	Sleep duration/ sleep quality	Screen time changes	Goal setting	Mother’s well-being
INFANT: Campbell 2008 (2008), Australia [3, 57]	No significant change	N/A	↓ non-core drink at 9 months ↓sweet and snack at 20 months	N/A	N/A	N/A	↓TV viewing time at 20 months	N/A	N/A
Carlsen 2013 (2010), Denmark [29]	No significant change	↑ exclusive and partial breastfeeding rates at 6 months	N/A	N/A	N/A	N/A	N/A	N/A	N/A
PRIMROSE: Doring 2014 (2014), Sweden [30, 58]	No significant change	N/A	↑vegetables, fruits and fish; ↓sugary drinks, french fries at 4 years of age	N/A	No significant change	N/A	N/A	N/A	N/A
Franco-Antonio 2018 (2018), Spain [31]	N/A	↑Exclusive Breast Feeding (EBF) duration and any BF at 6 months	N/A	N/A	N/A	N/A	N/A	No change	N/A
MumBubConnect: Gallegos 2014 (2010), Australia [32]	N/A	↑in EBF rates	N/A	N/A	N/A	N/A	N/A	N/A	N/A
Gibby 2019 (2019), Hawai’I and Puerto Rico [33]	No significant change	No significant change	No significant change	N/A	N/A	N/A	N/A	N/A	N/A
Steps to Growing Up Healthy: Gorin 2014 (2014), USA [34, 59]	↓BMI percentile	N/A	↓ juice consumption, whole milk consumption at end of the intervention	N/A	No significant change	N/A	No significant change	No change	N/A
Healthy Habits, Happy Homes: Haines 2013 (2011), USA [35]	↓ BMI	N/A	N/A	N/A	N/A	↑sleep duration	↓TV viewing on weekend days	N/A	N/A
Hannan 2012 (2012), USA [36]	Healthier weight gain	N/A	N/A	N/A	N/A	N/A	N/A	N/A	N/A
Harris-Luna 2018 (2018), USA [37]	N/A	↑EBF	N/A	N/A	N/A	N/A	N/A	N/A	N/A
M528: Hmone 2017 (2015), Myanmar [38]	N/A	↑ EBF rate at 6 months	↓ bottle feeding, early introduction of complementary food	N/A	N/A	N/A	N/A	N/A	N/A
Healthy Babies: Horodynski 2011 (2011), USA [39]	N/A	Not published	Not published	N/A	N/A	N/A	N/A	N/A	N/A
Jiang 2014 (2014), China [40]	No significant change	↑ EBF rate at 6 months	No significant change	N/A	N/A	N/A	N/A	N/A	N/A
The Baby Milk Trial: Lakshman 2015 (2011), UK [41]	↓ weight gain at 6 months and at 12 months	N/A	No significant change	N/A	N/A	N/A	N/A	N/A	N/A
Growing Healthy: Laws 2018 (2015), Australia [42]	No significant change	No significant change	No significant change	N/A	N/A	N/A	N/A	N/A	N/A
Smart Moms: Nezami 2018 (2014), USA [43]	No significant change	N/A	↓SSB/juice	N/A	N/A	N/A	N/A	↓mother’s weight	N/A
Patel 2018 (2010), India [44]	N/A	No significant change	N/A	N/A	N/A	N/A	N/A	N/A	N/A
WIC: Pugh 2010 (2003), USA [45]	N/A	↑any BF at 6 weeks and at 12 weeks	N/A	N/A	N/A	N/A	N/A	N/A	N/A
Tahir 2013 (2010), Malaysia [46]	N/A	↑EBF at 1 month, 4 months	N/A	N/A	N/A	N/A	N/A	N/A	N/A
Baby’s first bites: van der Veek 2019 (2019), Netherlands [47]	Outcomes not published	N/A	Outcomes not published	N/A	N/A	N/A	N/A	N/A	N/A
Mothers & Others: Wasser 2017 (2013), USA [48]	Outcomes not published	N/A	Outcomes not published	N/A	N/A	N/A	Outcomes not published	N/A	N/A
Healthy Beginnings: Wen 2007 (2007), Australia [2, 60–62]	↓BMI	↑BF at 6 and 12 months	↓Introdution to solids prior to 6 months	↓ age at which infants started tummy time and ↑daily practice of tummy time	No significant change	↑ mean nocturnal sleep duration	No significant change	N/A	N/A
CHAT: Wen 2017 (2017), Australia [1]	Outcomes not published	No significant change	↑higher odds of appropriate timing of introducing solids (telephone support): ↓bottle at bedtime (telephone and SMS support)	↑early start of tummy time (Telephone support)	Outcomes not published	Outcomes not published	No screen time (Telephone and SMS support)	Outcomes not published	Outcomes not published
Linked trial for HB: Wen 2019 (2019), Australia [19]	Ongoing	Ongoing	Ongoing	Ongoing	Ongoing	Ongoing	Ongoing	Ongoing	Ongoing

Over two-fifths (44%; 7 of 16) demonstrated an increase in breastfeeding duration, 47% (7 of 15) reported improved food habits in children. Changes in feeding habits included: reduction in non-core drink consumption at 9 months of children’s age [57], and reduction in juice consumption and sugary drinks at 4 years of children’s age [43, 58, 59] in the intervention group in comparison to the control. There were higher odds of appropriate timing of introduction of solids in the intervention group in comparison to the control group (at 6–7 months of children’s age) [60–62]. 67% (2 of 3) reported increased practice of “tummy time”, 20% (1 of 5) reported an increase in children’s duration of outdoor activities, 50% (2 of 4) reported an increase in sleep duration of children, and 43% (3 of 7) reported a decrease in TV viewing or screen time.

We also looked for commonalities between effectiveness of interventions and mode of delivery. Of the studies that showed improvements in behaviours related to childhood obesity, 53% (8 of 15) were delivered solely via telephone or text messages.

### Process evaluation

Eight studies (33%) had evidence of process evaluation or satisfaction measures [1, 3, 32, 33, 38, 40–42]. All eight studies quantitatively measured participant satisfaction at the time interventions were delivered. Qualitative interviews with trial participants were conducted by three studies during the intervention phase [63–65] and by five studies post-intervention [32, 33, 66–68], with only one study measuring perceptions of participants and recruiters during the recruitment phase [69]. Details of this analysis are shown in supplementary file 1. Our assessment of the qualitative studies against the COREQ criteria showed that all studies except one (that included a self-assessment against COREQ) had insufficient information (supplementary file 2). Hence, we assessed the studies against the SRQR criteria: six studies reported sufficient information (supplementary file 3).

Four of the eight studies were conducted in Australia, two were collaborative studies with Australia conducted in China and Myanmar, one study was in the UK and one in Hawai’i/Puerto Rico. Six studies measured BMIz or weight change of which one study noted a decrease in weight gain in comparison to the control. Three studies noted increased breastfeeding rates and three studies observed improved feeding habits in comparison to the control. Two studies that targeted screen time in children found a reduction in screen time in comparison to the control. One study that targeted a range of behaviours observed an earlier start of tummy time by participants in comparison to the control (Table 5).

Participants’ perceptions / satisfaction with the program during the intervention phase of the study were evaluated by three studies through in-depth interviews [65], qualitative interviews [64] and semi-structured qualitative interviews with a purposive sample of participants during intervention phase [63]. Five studies evaluated participants’ perceptions upon completion of the intervention or post-intervention period through semi-structured interviews [68], semi-structured telephone interviews with purposive sampling [67], qualitative interviews [66], a questionnaire with open-ended process evaluation questions [32] and an in-person exit interview [33]. Additional process evaluation components included examination of researchers’ diaries, field records, project meeting minutes [64], and interviews with participants and recruiters during the recruitment phase to assess facilitators and challenges in recruiting pregnant women to trials [69] (Table 6).

**Table 6:** Process evaluation of interventions

Trial name: First author publication year (year study commenced), Country (Reference #)	‘Process evaluation’ or satisfaction measurement	Evaluation components	Quantitative evaluation (including during or post-intervention)	Qualitative evaluation (including during or post-intervention)	Participant perceptions where evaluated
INFANT: Campbell 2008 (2008), Australia [3, 66]	Process evaluation through response on a 4-point scale - quantitative	At each session, participants were asked to rate usefulness and relevance of the program on a 4-point scale from “not at all useful/relevant” to “very useful/relevant.” (i.e., “How useful was the session overall?” and “How relevant was this session to you and your family?”)	Participants were asked to complete forms after each session and indicate usefulness and relevance of the program and components of each of the sessions (during intervention)	Qualitative interviews were conducted 3–5 months after the completion of the program (post-intervention)	• Preference for combination of delivery modes • Appealed to first-time mothers • Participants’ lack of time to participate due to return to work
Carlsen 2013 (2010), Denmark [29]	None specified	None specified	Participants were not asked to rate satisfaction	Participants were not interviewed	Not evaluated
PRIMROSE: Doring 2014 (2014), Sweden [30]	None specified	None specified	Participants were not asked to rate satisfaction	Participants were not interviewed	Not evaluated
Franco-Antonio 2018 (2018), Spain [31]	None specified	None specified	None specified	None specified	Not evaluated
MumBubConnect: Gallegos 2014 (2010), Australia [32]	Process evaluation	Frequency of text messages sent and responses received; number of telephone calls made by breastfeeding counsellor; Qualitative responses gathered via questionnaire to obtain women’s acceptability of service	Frequency of text messages sent and responses received; number of telephone calls made by breastfeeding counsellor (during intervention)	Qualitative responses gathered via post-intervention survey questionnaire to obtain women’s acceptability of service (post-intervention)	• Considered themselves well supported through participation in program
Gibby 2019 (2019), Hawai’I and Puerto Rico [33]	Satisfaction	Usefulness of text messages; how participants were influenced to change behaviours; and most and least liked messages; Satisfaction with the text messages delivered. Qualitative interviews at follow-up visits.	Most liked and least liked messages were rated by participants	At the follow-up visits, participants completed a qualitative interview regarding helpfulness of messages, ways in which receiving the messages influenced or changed feeding practices and overall feedback about receiving the messages. Responses to 6 open-ended questions (post-intervention)	• More likely to make changes if the content delivered aligned with their pre-existing beliefs • Level of engagement with programs fluctuated, based on their needs at a particular point in time and based on their child’s stage of development
Steps to Growing Up Healthy: Gorin 2014 (2014), USA [34]	Process evaluation stated in study protocol paper, no evidence of one being conducted	At the end of intervention period mothers asked to evaluate the program - helpfulness, components most useful, refer friend to program			Not evaluated
Healthy Habits, Happy Homes: Haines 2013 (2011), USA [35]	None specified	None specified			Not evaluated
Hannan 2012 (2012), USA [36]	None specified	None specified			Not evaluated
Harris-Luna 2018 (2018), USA [37]	None specified	None specified			Not evaluated
M528: Hmone 2017 (2015), Myanmar [38, 65]	Process evaluation	The process evaluation used both quantitative phone-based surveys and qualitative in-depth interviews.	Informal assessment of user experience, acceptability, feedback from participants via text messages (during intervention)	In-depth semi-structured interviews with a sub-sample to explore user experience, perception and acceptance (during intervention)	•Behaviour modification likely if information is from a credible source such as from health professionals
Healthy Babies: Horodynski 2011 (2011), USA [39]	Process evaluation stated in study protocol paper, no evidence of one being conducted	Proposal to conduct: Feasibility, fidelity, and educational effectiveness of interventions. Mothers’ satisfaction with the Healthy Babies intervention; Recruitment; retention;	Proposal to conduct only - not published	Proposal to conduct interviews - not published	Not evaluated
Jiang 2014 (2014), China [40, 64]	Process evaluation	A 3-phase process during planning and development	Baseline questionnaire survey to understand potential intervention approaches	Qualitative interviews with mothers midterm and at the end of the intervention	• Behaviour modification likely if information is from a credible source such as from health professionals • Delivery of interventions via text messages facilitated sharing of messages with family and friends • Lack of personalisation of contents in text messages
The Baby Milk Trial: Lakshman 2015 (2011), UK [41, 68]	Process evaluation	Parents’ satisfaction with different aspects of the intervention will be assessed at 6mo via questionnaire	Questionnaire at 6 months to assess parents’ satisfaction with intervention	Semi-structured interviews with sub-sample of intervention and control group participants and facilitators to explore barriers and facilitators	• All participants reported the trial participation as a positive experience • They shared various experiences of not getting external help, support, or information about formula-feeding • Most notably, the most positive outcome of the trial participation for the mothers, probably not captured in the trial’s quantitative outcome measures but a central finding in this qualitative study, was the personal and non-judgmental support they received for formula-feeding
Growing Healthy: Laws 2018 (2015), Australia [42, 67]	Process evaluation not specified. Acceptability measured	Assessment of App usage and App acceptability	Participant views	Qualitative follow-up interviews with parents	• Behaviour modification likely if information is from a credible source such as from health professionals • More likely to make changes if the content delivered aligned with their pre-existing beliefs • Level of engagement with programs fluctuated, based on their needs at a particular point in time and based on their child’s stage of development • Appealed to first-time mothers • Preference for a combination of delivery modes (eg., text messages, telephone calls, emails, push notifications, web, group sessions)
Smart Moms: Nezami 2018 (2014), USA [43]	None specified	None specified			Not evaluated
Patel 2018 (2010), India [44]	Process evaluation not specified	Process evaluation not specified	Not measured	None specified	Not evaluated
WIC: Pugh 2010 (2003), USA [45]	None specified	None specified	None specified	None specified	Not evaluated
Tahir 2013 (2010), Malaysia [46]	None specified	None specified	None specified	None specified	Not evaluated
Baby’s first bites: van der Veek 2019 (2019), Netherlands [47]	None specified	None specified	None specified	None specified	Not evaluated
Mothers & Others: Wasser 2017 (2013), USA [48]	None specified	None specified	None specified	None specified	Not evaluated
Healthy Beginnings: Wen 2007 (2007), Australia [2]	Process evaluation not specified	None specified	Questionnaires to participants only to evaluate infant feeding such as duration of breastfeeding, introduction of solids and healthy feeding practice	None specified	Not evaluated
CHAT: Wen 2017 (2017), Australia [1, 63, 69]	Process evaluation in protocol	Documentation of contact with families by intervention nurses; recruitment data barriers and enablers; study retention and intervention acceptability; interviews with participants to assess program satisfaction; identify emerging issues	Satisfaction questions administered at the 6-month and 12-month surveys	Interviews with participants to assess program satisfaction; identify emerging issues (during intervention)	• Consented to participate due to convenience of receiving interventions via telephone calls or text messages. • Behaviour modification likely if information is from a credible source such as from health professionals • Delivery of interventions via text messages facilitated sharing of messages with family and friends • Level of engagement with programs fluctuated, based on their needs at a particular point in time and based on their child’s stage of development • Preference for a combination of delivery modes (eg., text messages, telephone calls, emails, push notifications, web, group sessions) • Participation via telephone calls and by text messages was convenient • Appealed to first-time mothers • Considered themselves well supported through participation in program • Participants’ lack of time to participate due to return to work • Lack of personalisation of contents in text messages • High expectations placed on them as mothers
Linked trial for HB: Wen 2019 (2019), Australia [19]	Process evaluation of telephone contact with mothers (stated in study protocol, no evidence since study was ongoing)	Thematic analysis of participants’ responses (de-identified) will be evaluated retrospectively	Ongoing	Ongoing	Ongoing

Process evaluation of the recruitment phase of the studies indicated that participants consented to participate due to the convenience of the delivery mode via telephone or text messages [69]. Evaluation of participants’ experience indicated that participants were likely to modify behaviour if they received information from a credible source such as from health professionals [63–65, 67]. Delivery of interventions via text messages facilitated sharing of messages with family and friends [63, 64]. Participants were more likely to adhere to recommendations and change behaviours if the content delivered aligned with their pre-existing beliefs [33, 67]. Participants’ levels of engagement with programs fluctuated based on their needs and their available time at later stages of their children’s development [33, 63, 67]. Participation via telephone and by text messages was convenient to participants [63], and participants expressed preferences for receiving interventions through a combination of non-face-to-face delivery modes including but not limited to text messages, telephone, emails, Web and push notifications [63, 67]. The programs were more appealing to first-time caregivers in comparison to those who cared for previous children [63, 66, 67] and participants considered themselves well supported through participation [32, 63]. Some barriers to participation included: lack of personalisation of text messages [63, 64]; participants’ lack of time due to return to work [63, 66]; and where participants perceived that high expectations were placed on them as mothers [63]. The process evaluation findings are represented in Table 6.

## Discussion

### Key findings

The objective of this systematic review was to explore the acceptability of the interventions to stakeholders through process evaluation of early childhood obesity prevention studies. Of the 24 eligible studies that delivered interventions via telephone or text messages, only one-third of studies (*n* = 8) examined stakeholder perceptions, with all of these studies focussing on the satisfaction / acceptability of the interventions that were delivered to participants. We found no evidence of evaluation of perceptions of other key stakeholders including those who delivered the interventions or health managers or policymakers, and no evidence of other process evaluation measures such as reach or fidelity.

Process evaluation findings highlight participants’ appreciation of the convenience of receiving interventions via telephone or text messages [63, 69], and the importance of delivering interventions from credible sources for participants’ compliance with interventions and behaviour changes [63–65, 67]. Level of engagement in a program was not dependent on the mode of delivery but was dictated by participants’ needs and on their children’s developmental stage [33, 63, 67]. Although participants perceived telephone or text messages as convenient, they expressed preference to be able to receive interventions through a combination of one or more delivery methods, namely, telephone, text messages, Web, apps with optional face-to-face [63, 66, 67]. Participants highlighted the co-benefits they received, such as early identification of any issues (clinical, social or similar needs) and referral to appropriate services. Participants considered themselves well supported [32, 63], with first-time caregivers considering the programs more valuable than those who had previous children [63, 66, 67]. Participants expressed some barriers to participation such as lack of personalisation of content in text messages [63, 64], lack of time due to return to work (irrespective of the mode of delivery) [63, 66] and a perception that high expectations were placed on them as mothers [63].

The growth in childhood obesity prevention interventions delivered by telephone/text messages is shown by the large proportion of studies conducted in the last decade. Similar to previous systematic reviews of childhood obesity prevention interventions [25, 70], several outcomes were measured including BMIz or weight gain, breastfeeding, solid feeding/food habits, tummy time, play time/physical activity, sleep duration/sleep quality, screen time/TV viewing, goal-setting and mother’s well-being. Less than one-quarter (23%; 3 of 13) of the studies that measured outcomes for weight and BMIz reported a statistically significant decrease in weight gain or a lower BMIz score in comparison to the control [25, 70, 71], while over three-fifths (63%; 15 of 24) of all studies in this review showed improvement in one or more behaviours related to childhood obesity prevention. Previous reviews have reported inconsistent outcomes for behaviour changes [25, 70]. Studies that were included in this review provided interventions for “tummy time” and sleep duration that were not included in previous reviews. These outcomes suggest that while it is more difficult to change weight outcomes such as BMIz, interventions delivered by telephone can be effective in supporting behaviours important for the prevention of obesity.

Delivery of interventions remotely via telephone has been proven to be more cost-effective [72]. Although text only studies would be the most cost-effective method of delivery, there was limited evidence in this review, with just three studies delivering interventions solely via text messages for breastfeeding of which two demonstrated an increase in exclusive breastfeeding. The average retention rates for studies delivered with and without a face-to-face component were both 85%. This may suggest that interventions delivered remotely via telephone or text messages have the potential to reach, attract and retain equal or a greater number of participants than those delivered via face-to-face modes. This implies that childhood obesity prevention interventions delivered via telephone or text messages have the potential to be more cost-effective and have equal or greater reach than interventions that include a face-to-face component.

### Comparison with prior reviews

Previous systematic reviews of early childhood obesity (0–5 years of age) prevention trials have not examined process evaluation or participant involvement but have recommended inclusion of these components for improved quality and relevance [25]. Although the focus of previous reviews was not on delivery of interventions via telephone or text messages, multiple modes of traditional delivery methods were employed [73] and the reviews recommended exploring intervention delivery via low cost methods such as telephone and the internet [71]. Three of the previous reviews examined delivery of interventions exclusively by healthcare professionals e.g., research nurses, lactation consultants, psychologists and social workers [25, 70, 74]. Similarly, in almost four-fifths of the studies in this review, interventions were delivered by health professionals such as nurses, midwives, health educators, or dietitians; and in one-fifth, interventions were delivered via automated text messages, apps or online.

Systematic reviews of obesity prevention interventions delivered to older children and adolescents (12–24 years of age) using mobile technologies have noted heterogeneity in research design and in the interventions delivered [75–77]. These reviews observed a small number of studies that delivered interventions to adolescents and young adults via telephone, text messages or mobile apps. Very limited or post hoc process evaluation studies were noted [76] and research in this area was considered to be in its infancy with further research required to elucidate effectiveness [75, 76].

Previous reviews have not reported on process evaluation literature but noted its potential value [26, 71, 76]. Process evaluation findings in this review demonstrate that participants valued and trusted interventions delivered from credible sources, hence intervention deliverers are crucial to the acceptability of interventions. Thorough reporting of recruitment and training of intervention deliverers is important in replicating intervention effects during scale up [26, 71]. This review demonstrates limited evidence of evaluation of participants’ perceptions and a lack of evidence that existing studies have examined the perceptions of intervention deliverers, health professionals and policymakers.

### Public health implications

Evidence gathered through process evaluation of trials contribute crucial knowledge to refinement of interventions and programs prior to their replication and scale up [78, 79]. Additionally, process evaluation of trials facilitates integration of qualitative and quantitative methods that yields rich detail about study outcomes that neither method could achieve alone [78, 80]. Although process evaluation has been in existence for over two decades, only one-third of the studies in this review had evidence of process evaluation or satisfaction measurement, demonstrating the limited number of studies that conducted process evaluation to measure stakeholder perception. The findings from this review provide important insights for researchers about the importance of conducting process evaluation alongside trials to explore the perceptions of stakeholders in addition to evaluating effectiveness of interventions. While outcome measures of childhood obesity prevention interventions are indicative of the success of programs delivered to caregivers with young children, a key component of the success is attributed to the acceptability of, and compliance with the program by its participants.

Although process evaluation often takes a back seat to impact evaluation, information about stakeholders’ perceptions and how a program is implemented, makes it easier to understand why participants did or did not gain some benefit from participating in the program [81]. Stakeholder feedback obtained as a result of process evaluation is important for modifying and improving interventions to enhance engagement, retention and effectiveness of programs prior to scale up [78, 81]. In circumstances where comprehensive process evaluation is not feasible due to limited resources or time pressures in trial environments, at a minimum, evaluating the perceptions of participants, intervention deliverers, health managers and policymakers during or immediately after intervention delivery is warranted [78].

### Review strengths and limitations

This systematic review has a number of strengths. The scope and search for this systematic review was comprehensive and conducted in accordance with the Preferred Reporting Items for Systematic Reviews and Meta-Analysis (PRISMA) standardised reporting guidelines and checklist [27]. A protocol was developed prior to the review process and registered with PROSPERO. Eligible studies were identified using the Population, Intervention, Comparison, Outcomes (PICO) framework [28]. Titles and abstracts of references were independently screened by two reviewers in Covidence. Data were gathered and analysed similar to that described in the template for intervention description and replication (TIDieR) [49]. Risk of bias for all eligible studies was independently assessed by two reviewers using the Cochrane Collaboration tool for assessing risk of bias [53]. Qualitative studies were assessed using the COREQ and SRQR tools [55, 56], our assessment demonstrated lack of evidence of elements described in the tools. One recommendation is for qualitative studies to include self-assessment against a standard tool.

However, this review only included peer-reviewed papers published in English. Therefore, we may have missed peer-reviewed literature published in other languages. Despite our best efforts to obtain further information from study investigators of ongoing trials, this review was not able to include information on those ongoing or unpublished studies, and two studies did not conduct process evaluation as planned. The main limitation of this review stems from the small number of studies that conducted and reported process evaluation data, limiting our ability to describe effective engagement and retention approaches for scale up of programs.

## Conclusion

Of the 24 studies included in this review, only one-third reported process evaluation to measure perceptions of participants. Evaluation of participants’ experiences during recruitment and intervention phases demonstrated the potential for childhood obesity prevention interventions to be delivered conveniently via telephone or inexpensively via text messages. Interventions delivered remotely via telephone or text messages have the potential to reach, attract and retain equal or a greater number of participants than those delivered via face-to-face methods. While outcomes for weight varied, many of the studies in this review showed improvements in behaviours related to childhood obesity. This review shows that the conduct of process evaluation alongside trials is uncommon, future studies should build in process evaluation alongside effectiveness measurements to provide important insight into intervention reach, acceptability and to inform scale up.

## Supplementary Information

**Additional file 1.** Supplementary file 1 Assessment of evidence for process evaluation or satisfaction within studies. Supplementary file 2 Assessment of qualitative studies against the COREQ tool. Supplementary file 3 Assessment of qualitative studies against the SRQR tool. Supplementary file 4 PRISMA checklist.

## Data Availability

All data generated or analysed during this study are included in this published article and its supplementary information files.

## Abbreviations

RCT: Randomised controlled trial
BMI: Body mass index
BMIz: Age and sex standardized body mass index z-score
PICO: Population, Intervention, Comparison, Outcomes
SRQR: Standards for Reporting Qualitative Research
PRISMA: Preferred Reporting Items for Systematic Reviews and Meta-Analysis
PROSPERO: Prospective Register for Systematic Reviews
TIDieR: Template for intervention description and replication
