# Supplementary material for: Process evaluations of early childhood obesity prevention interventions delivered via telephone or text messages: a systematic review
Source: Int J Behav Nutr Phys Act. 2021 Jan 9;18:10. doi: 10.1186/s12966-020-01074-8 (PMC7796572; doi:10.1186/s12966-020-01074-8)
Supplement: Supplementary file 1 — Additional file 1. Supplementary file 1 Assessment of evidence for process evaluation or satisfaction within studies. Supplementary file 2 Assessment of qualitative studies against the COREQ tool. Supplementary file 3 Assessment of qualitative studies against the SRQR tool. Supplementary file 4 PRISMA checklist. [file 12966_2020_1074_MOESM1_ESM.zip › Supplementary file 3_ESM.docx]

Supplementary file 3 Assessment of qualitative studies against the SRQR tool
